# Lipoprotein(a) and Long-Term Plaque Progression, Low-Density Plaque, and Pericoronary Inflammation

**DOI:** 10.1001/jamacardio.2024.1874

**Published:** 2024-07-17

**Authors:** Nick S. Nurmohamed, Emilie L. Gaillard, Shant Malkasian, Robin J. de Groot, Shirin Ibrahim, Michiel J. Bom, Yannick Kaiser, James P. Earls, James K. Min, Jeffrey Kroon, R. Nils Planken, Ibrahim Danad, Alexander R. van Rosendael, Andrew D. Choi, Erik S.G. Stroes, Paul Knaapen

**Affiliations:** 1 Department of Cardiology, Amsterdam UMC, Vrije Universiteit Amsterdam, Amsterdam, the Netherlands; 2 Department of Vascular Medicine, Amsterdam UMC, University of Amsterdam, Amsterdam, the Netherlands; 3 Division of Cardiology, The George Washington University School of Medicine, Washington, DC; 4 Department of Radiological Sciences, Medical Sciences I, University of California, Irvine, California; 5 Cleerly, Denver, Colorado; 6 Department of Experimental Vascular Medicine, Amsterdam UMC, University of Amsterdam, Amsterdam, the Netherlands; 7 Laboratory of Angiogenesis and Vascular Metabolism, VIB-KU Leuven Center for Cancer Biology, Leuven, Belgium; 8 Department of Radiology and Nuclear Medicine, Amsterdam UMC, Universiteit van Amsterdam, Amsterdam, the Netherlands; 9 Department of Cardiology, University Medical Center Utrecht, Utrecht, the Netherlands; 10 Department of Cardiology, Leiden University Medical Center, Leiden, the Netherlands

## Abstract

**Importance:**

Lipoprotein(a) (Lp[a]) is a causal risk factor for cardiovascular disease; however, long-term effects on coronary atherosclerotic plaque phenotype, high-risk plaque formation, and pericoronary adipose tissue inflammation remain unknown.

**Objective:**

To investigate the association of Lp(a) levels with long-term coronary artery plaque progression, high-risk plaque, and pericoronary adipose tissue inflammation.

**Design, Setting, and Participants:**

This single-center prospective cohort study included 299 patients with suspected coronary artery disease (CAD) who underwent per-protocol repeated coronary computed tomography angiography (CCTA) imaging with an interscan interval of 10 years. Thirty-two patients were excluded because of coronary artery bypass grafting, resulting in a study population of 267 patients. Data for this study were collected from October 2008 to October 2022 and analyzed from March 2023 to March 2024.

**Exposures:**

The median scan interval was 10.2 years. Lp(a) was measured at follow-up using an isoform-insensitive assay. CCTA scans were analyzed with a previously validated artificial intelligence–based algorithm (atherosclerosis imaging-quantitative computed tomography).

**Main Outcome and Measures:**

The association between Lp(a) and change in percent plaque volumes was investigated in linear mixed-effects models adjusted for clinical risk factors. Secondary outcomes were presence of low-density plaque and presence of increased pericoronary adipose tissue attenuation at baseline and follow-up CCTA imaging.

**Results:**

The 267 included patients had a mean age of 57.1 (SD, 7.3) years and 153 were male (57%). Patients with Lp(a) levels of 125 nmol/L or higher had twice as high percent atheroma volume (6.9% vs 3.0%; *P* = .01) compared with patients with Lp(a) levels less than 125 nmol/L. Adjusted for other risk factors, every doubling of Lp(a) resulted in an additional 0.32% (95% CI, 0.04-0.60) increment in percent atheroma volume during the 10 years of follow-up. Every doubling of Lp(a) resulted in an odds ratio of 1.23 (95% CI, 1.00-1.51) and 1.21 (95% CI, 1.01-1.45) for the presence of low-density plaque at baseline and follow-up, respectively. Patients with higher Lp(a) levels had increased pericoronary adipose tissue attenuation around both the right coronary artery and left anterior descending at baseline and follow-up.

**Conclusions and Relevance:**

In this long-term prospective serial CCTA imaging study, higher Lp(a) levels were associated with increased progression of coronary plaque burden and increased presence of low-density noncalcified plaque and pericoronary adipose tissue inflammation. These data suggest an impact of elevated Lp(a) levels on coronary atherogenesis of high-risk, inflammatory, rupture-prone plaques over the long term.

## Key Points

**Question:**

Is lipoprotein(a) (Lp[a]) associated with adverse long-term plaque progression in patients at risk for atherosclerotic cardiovascular disease?

**Findings:**

In this serial coronary computed tomography angiography imaging study with an interscan interval of 10 years, Lp(a) was positively associated with coronary plaque burden at baseline. Adjusted for other risk factors, every doubling of Lp(a) resulted in an additional 0.32% increment in percent atheroma volume for every 10 years of follow-up.

**Meaning:**

These findings show that higher Lp(a) levels are associated with increased progression of coronary plaque burden.

## Teaser

This study investigates the impact of lipoprotein(a) levels on long-term coronary artery plaque progression, high-risk plaque, and pericoronary adipose tissue inflammation.

## Introduction

Lipoprotein(a) (Lp[a]) is an important causal risk factor for the occurrence of atherosclerotic cardiovascular disease events. Supporting evidence has been provided by large genetic and observational studies in more than 500 000 patients, which have also shown Lp(a) is predominantly (more than 90%) genetically determined and, thus, remains a stable risk factor throughout life.^1^ Despite recent guidelines that have recommended measuring Lp(a) at least once in every adult, long-term effects on plaque development remain unknown.^1,2^

In contrast with low-density lipoprotein (LDL) cholesterol, preclinical studies have illustrated that the atherogenicity of Lp(a) is predominantly caused by carried oxidized phospholipids initiating proinflammatory and procalcific pathways at the plaque level.^3,4^ Patients with elevated Lp(a) levels have arterial wall inflammation and increased migration of monocytes into the atherosclerotic plaque.^4^ We recently showed that Lp(a) might be associated with a high-risk plaque phenotype in a small cohort of 160 patients undergoing repeat coronary computed tomography angiography (CCTA) imaging after 1 year.^5^ However, the effect of Lp(a) on long-term longitudinal progression of coronary plaque has not been investigated to date. This study set out to investigate the association of Lp(a) levels with long-term coronary artery plaque progression using a cohort of 299 patients who underwent serial CCTA imaging with an interscan interval of 10 years.

## Methods

### Patient Population

This prospective long-term serial CCTA study was performed in a cohort of patients who underwent baseline CCTA imaging for suspected stable coronary artery disease (CAD) between 2008 and 2014 at the Amsterdam University Medical Center (Amsterdam, the Netherlands).^6,7^ At the time of baseline imaging, patients had no history of CAD. Per the research protocol, patients were invited for repeat CCTA imaging, regardless of symptoms or history. The study complied with the Declaration of Helsinki. The current follow-up study was separately approved by the local ethics committee and participants provided separate informed consent for the follow-up CCTA study. Of 465 patients considered for follow-up imaging, 38 patients did not meet the eligibility criteria, while 128 opted out for repeat imaging (eFigure 1 in Supplement 1). Patients who did not undergo follow-up imaging were older and more likely to be female (eTable 1 in Supplement 1). A total of 299 patients underwent serial CCTA imaging, of whom 90 patients underwent percutaneous coronary intervention (n = 61) or coronary artery bypass grafting (n = 32) following baseline imaging or during follow-up (3 patients underwent both). The 32 patients who underwent coronary artery bypass grafting were excluded from the present analysis, resulting in a final study population of 267 patients (eFigure 1 in Supplement 1).

### Lp(a) Measurement

Lp(a) plasma concentrations were measured in nmol/L by an isoform-insensitive, second-generation assay (Roche Diagnostics) performed at follow-up imaging. Since multiple studies have shown that Lp(a) plasma levels in adults are stable for more than 90% over a lifetime,^1^ Lp(a) levels were considered equal throughout the study. For the current study, Lp(a) levels of 125 nmol/L or higher were considered abnormal.^1^

### CCTA Imaging

At baseline imaging, all patients underwent combined coronary artery calcium scoring (CACS) and CCTA using 64-slice or higher CCTA scanners from the same manufacturer (Philips Healthcare), as described previously (eMethods in Supplement 1).^8,9^ At follow-up, patients also underwent combined CACS and CCTA using a third-generation dual-source CT scanner (SOMATOM Force; Siemens; eMethods in Supplement 1).

### Atherosclerosis Imaging Quantitative Computed Tomography Analysis

An artificial intelligence–based software approach was used to analyze the CCTA images (atherosclerosis imaging quantitative computed tomography; Cleerly; eMethods in Supplement 1).^10^ Coronary plaque volume was normalized to vessel volume to account for variation in coronary artery volume, calculated as plaque volume / vessel volume × 100%. These normalized volumes were reported as percent atheroma volume (PAV), percent noncalcified plaque volume, and percent calcified plaque volume. Furthermore, the presence of low-density noncalcified plaque was assessed. Increased pericoronary adipose tissue attenuation (PCATa) was defined as having a PCATa above the scanner-specific threshold, which was determined as the median value in all patients undergoing CCTA on the particular scanner and settings.^8^

In the serial analysis, when impaired image quality was present due to motion, poor opacification, beam hardening, or other artifact in a certain vessel, vessels were excluded both at baseline and follow-up imaging analysis. Coronary artery vessels with stent placement at follow-up were also excluded from baseline and follow-up imaging analysis, ensuring a 1-to-1 comparison. In total, 10.5% of the coronary vessels was excluded due to impaired image quality or stent placement. An additional sensitivity analysis was performed imputing the plaque volumes in these vessels.

### Study Outcomes

The coprimary outcomes were defined as the absolute change in PAV and percent noncalcified plaque volume, which were calculated subtracting baseline values from follow-up values. Secondary outcomes were defined as change in calcified plaque volume, as well as presence of low-density plaque and increased pericoronary attenuation, at baseline and follow-up imaging.

### Statistical Analysis

The association between Lp(a) and plaque volumes over time was assessed using linear mixed-effect regression models with random intercept to account for within-patient clustering. In these models, an interaction term between time and Lp(a) levels, as well as covariates, was included to assess the effect of Lp(a) on plaque progression. The difference in plaque volumes and change in plaque volumes was graphically displayed over time using the estimates from the univariate linear mixed models for the 10th, 50th, and 90th percentile. In the sensitivity analysis with imputation, vessels with missing plaque volumes at baseline or follow-up were imputed using plaque volumes at the other time point and clinical characteristics using multiple imputation with chained equations. The association between Lp(a) levels, low-density noncalcified plaque, and PCATa was assessed using logistic regression models, separate for baseline and follow-up imaging. In the multivariable linear and logistic regression models, Lp(a) values were log2 transformed prior to the analyses due to the right-skewed distribution, ie, every 1-point increase reflected a doubling in Lp(a) levels. The multivariable linear mixed-effects and logistic regression models were adjusted for age, sex, and clinical risk factors (history of hypertension, history of hypercholesterolemia [prior or baseline total cholesterol 6.5 mmol/L (approximately 250 mg/dL)] or higher), LDL cholesterol, high-density lipoprotein cholesterol, triglycerides, systolic blood pressure, estimated glomerular filtration rate, type 2 diabetes, body mass index, smoking status, family history of CAD, and statin intensity^11^ at baseline and follow-up.

Data are presented as mean (SD) for normally distributed variables or median with interquartile range (IQR) for nonnormally distributed data. Categorical variables are expressed as absolute numbers and percentages. Independent-sample *t* tests, Wilcoxon tests, Mann-Whitney *U *tests, and Kruskal-Wallis tests were used where appropriate. All statistical analyses were performed using RStudio software version 4.0.3 (R Foundation).

## Results

### Patient Characteristics

The 274 patients had a mean age at baseline of 57 (SD, 7) years and 153 were male (57%) (Table 1). Patients experienced a range of symptoms comprising typical angina (85 [32%]), atypical angina (96 [37%]), and nonspecific chest pain (82 [31%]) as reason for referral for baseline CCTA imaging. At baseline, median PAV was 3.3% (IQR, 0.9-9.9), reflecting a total plaque volume of 96.2 (IQR, 27.1-274.2) mm^3^. The median calcified plaque volume was 0.5% (IQR, 0.0-3.2), and 12.6 (IQR, 0.0-80.8) mm^3^, while noncalcified plaque volume was 2.6% (IQR, 0.9-6.7) and 65.5 (IQR, 23.0-183.2) mm^3^. The median interval between baseline and follow-up imaging was 10.2 (IQR, 8.8-11.2) years.

**Table 1.** Baseline Characteristics

	No. (%)			
Characteristic	Overall (n = 267)	Lp(a) <125 nmol/L (n = 206)	Lp(a) ≥125 nmol/L (n = 61)	*P* value
Age at baseline, y, mean (SD)	57.1 (7.3)	56.6 (7.1)	59.1 (7.6)	.02
Sex				
Female	114 (43)	86 (42)	92 (46)	.57
Male	153 (57)	120 (58)	33 (54)	
Hypertension	111 (42)	89 (43)	22 (36)	.32
Systolic blood pressure, mm Hg, mean (SD)	138 (19)	139 (20)	137 (19)	.47
Hypercholesterolemia	96 (36)	65 (32)	31 (51)	.01
LDL cholesterol, mg/dL, mean (SD)	101 (39)	101 (38)	103 (41)	.89
HDL cholesterol, mg/dL, mean (SD)	56 (21)	56 (21)	55 (18)	.84
Triglycerides, mg/dL, median (IQR)	124 (84-186)	124 (89-186)	106 (80-151)	.10
Lipoprotein(a), nmol/L, median (IQR)	25 (8-111)	17 (7-42)	209 (149-273)	NA
BMI, mean (SD)	26.9 (4.3)	27.0 (4.5)	26.7 (3.3)	.94
eGFR, ml/min/1.73 m^2^, median (IQR)	97 (83-112)	95 (83-111)	102 (82-113)	.47
Type 2 diabetes	44 (16)	35 (17)	9 (15)	.68
Family history of CAD	148 (55)	115 (56)	33 (54)	.81
Smoking history	80 (30)	62 (30)	18 (30)	.93
Reason for referral				
Nonspecific chest pain	82 (31)	63 (31)	19 (31)	.97
Atypical angina	96 (37)	73 (36)	23 (38)	
Typical angina	85 (32)	66 (33)	19 (31)	
Statin use				
No statin	102 (38)	87 (42)	15 (25)	.01
Low intensity	3 (1.1)	1 (0.5)	2 (3.3)	
Moderate intensity	133 (50)	100 (49)	33 (54)	
High intensity	29 (11)	18 (9)	11 (18)	
Aspirin use	185 (70)	141 (70)	44 (72)	.73
β-Blocker use	154 (59)	119 (59)	35 (57)	.83
Calcium antagonist use	62 (24)	47 (23)	15 (25)	.83
Baseline CAD-RADS stage				
0	10 (3.7)	9 (4.4)	1 (1.6)	.11
1	135 (51)	111 (54)	24 (39)	
2	45 (17)	33 (16)	12 (20)	
3	35 (13)	22 (11)	13 (21)	
4/5	42 (16)	31 (15)	11 (18)	
Presence of obstructive stenosis	77 (29)	53 (26)	24 (39)	.04
Total plaque volume, mm^3 ^, median (IQR)	96.2 (27.1-274.2)	80.1 (22.5-223.1)	152.5 (38.8-403.9)	.01
Noncalcified plaque volume, mm^3 ^, median (IQR)	65.5 (23.0-183.2)	63.0 (19.3-156.7)	106.3 (31.7-234.9)	.03
Calcified plaque volume, mm^3 ^, median (IQR)	12.6 (0.0-80.8)	8.9 (0.0-67.2)	32.7 (0.7-137.8)	.003
Atheroma volume, %, median (IQR)	3.3 (0.9-9.9)	2.9 (0.8-9.1)	5.8 (1.8-13.2)	.01
Noncalcified plaque, % volume, median (IQR)	2.6 (0.9-6.7)	2.2 (0.8-5.7)	4.0 (1.4-8.2)	.03
Calcified plaque volume, %, median (IQR)	0.5 (0.0-3.2)	0.3 (0.0-2.4)	1.6 (0.3-5.3)	.003

Abbreviations: BMI, body mass index (calculated as weight in kilograms divided by height in meters squared); CAD, coronary artery disease; CAD-RADS, coronary artery disease reporting and data system; eGFR, estimated glomerular filtration rate; HDL, high-density lipoprotein; IQR, interquartile range; LDL, low-density lipoprotein; Lp(a), lipoprotein(a); NA, not applicable.

### Association Between Lp(a), Coronary Plaque Burden, and Plaque Progression

Compared with patients with Lp(a) levels lower than 125 nmol/L, patients with Lp(a) levels of 125 nmol/L or higher had a higher PAV (5.8; IQR, 1.8-13.2 vs 2.9; IQR, 0.8-9.1; *P* = .01; Table 1), higher percent noncalcified plaque volume (4.0; IQR, 1.4-9.0 vs 2.3; IQR, 0.8-6.5; *P* = .03), and higher percent calcified plaque volume at baseline (1.6; IQR, 0.0-5.3 vs 0.3; IQR, 0.0-2.4; *P* = .003). Patients with high Lp(a) levels also had increased plaque progression between baseline and follow-up compared with patients with low Lp(a) levels. The absolute change in PAV in patients with Lp(a) levels of 125 nmol/L or higher was 3.6 (IQR, 1.2-7.8) compared with 1.6 (IQR, 0.3-5.4) in patients with Lp(a) levels lower than 125 nmol/L (*P* = .004). For percent noncalcified plaque volume, the absolute change was 1.1 (IQR, 0.2-2.5) in patients with Lp(a) levels of 125 nmol/L or higher, while the absolute change was 0.5 (IQR, 0.0-2.4) in patients with Lp(a) levels lower than 125 nmol/L (*P* = .01). The absolute change in percent calcified plaque volume was 2.2 (IQR, 0.2-4.2) in patients with Lp(a) levels of 125 nmol/L or higher compared with 0.7 (IQR, 0.0-2.6) in patients with Lp(a) levels lower than 125 nmol/L (*P* = .004).

**Figure 1.**
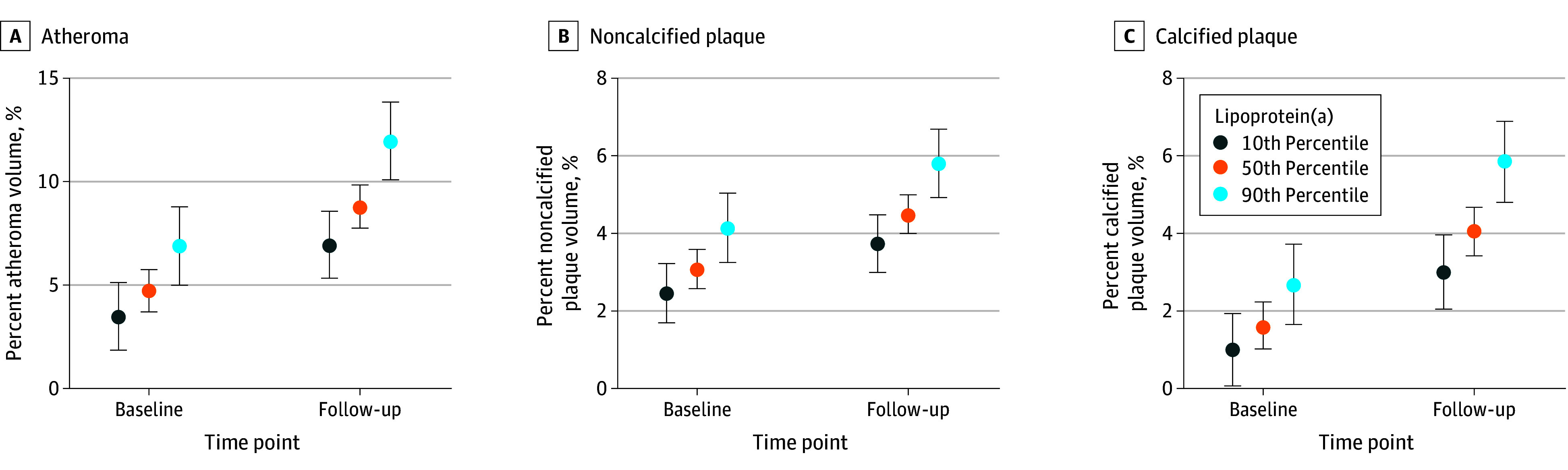
Association of Lipoprotein(a) (Lp[a]) With Plaque Burden and Coronary Computed Tomography Angiography (CCTA) Plaque Progression During Follow-Up The whiskers show 95% CIs from the unadjusted linear mixed-effect models for the 10th (7 nmol/L), 50th (25 nmol/L), and 90th (221 nmol/L) percentiles of Lp(a) in this study for percent atheroma volume (A), percent noncalcified plaque volume (B), and percent calcified plaque volume (C) at baseline and follow-up (10.2 years).

Lp(a) was associated with plaque volumes at baseline and follow-up in the univariate analysis (eFigure 2 in Supplement 1). In the linear mixed-effects model adjusted for clinical risk factors, every doubling of Lp(a) was associated with a 0.72% (95% CI, 0.23-1.21) higher PAV at baseline (*P* = .01; Table 2). Every doubling of Lp(a) resulted in an additional 0.32% (95% CI, 0.04-0.60) increment in PAV for every 10 years of follow-up (*P* = .03; Figure 1). In the model with percent noncalcified plaque volume, every doubling of Lp(a) was associated with a 0.38% (95% CI, 0.11-0.66) higher percent noncalcified plaque volume at baseline (*P* = .003; Table 2). Every doubling of Lp(a) resulted in an estimated 0.08% (95% CI, −0.08 to 0.23) increment in percent noncalcified plaque volume for every 10 years of follow-up (*P* = .33; Figure 1). In the adjusted linear mixed-effects model with percent calcified plaque volume, every doubling of Lp(a) was associated with a 0.34% (95% CI, 0.04-0.63) higher percent calcified plaque volume at baseline (*P* = .03; Table 2). Every doubling of Lp(a) was associated with an additional 0.22% (95% CI, 0.00-0.45) increment in percent calcified plaque volume during follow-up (*P* = .05; Figure 1). The findings were directionally similar in the sensitivity analysis with multiple imputation (eTable 2 in Supplement 1).

**Table 2.** Unadjusted and Adjusted Linear Mixed Models for the Association Between Lipoprotein(a) (Lp[a]), Follow-Up Time, and Plaque Volume

Volume type, %	Unadjusted β (95% CI)	*P* value	Adjusted β (95% CI)	*P* value
**Atheroma**				
Lp(a), per doubling	0.68 (0.14-1.22)	.01	0.72 (0.23-1.21)	.01
Follow-up time, per 10 y	2.48 (0.79-4.17)	.004	NA	NA
Lp(a) × follow-up time	0.32 (0.00-0.63)	.05	0.32 (0.04-0.60)	.03
**Noncalcified plaque**				
Lp(a), per doubling	0.34 (0.07-0.60)	.01	0.38 (0.11-0.66)	.003
Follow-up time, per 10 y	1.04 (0.23-1.86)	.01	NA	NA
Lp(a) × follow-up time	0.08 (−0.08 to 0.23)	.33	0.09 (−0.06 to 0.24)	.26
**Calcified plaque**				
Lp(a), per doubling	0.34 (0.02-0.65)	.04	0.34 (0.04-0.63)	.03
Follow-up time, per 10 y	1.31 (0.10-2.51)	.03	NA	NA
Lp(a) × follow-up time	0.23 (0.01-0.46)	.04	0.22 (0.00-0.45)	.05

Abbreviation: NA, not applicable.

### Association Between Lp(a) Levels, High-Risk Plaque Phenotype, and Pericoronary Adipose Tissue Inflammation

**Figure 2.**
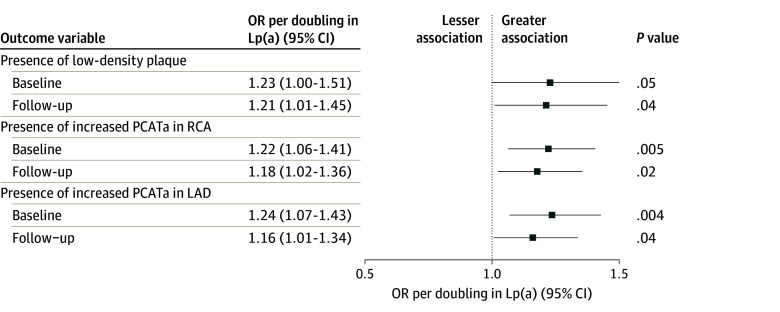
Association of Lipoprotein(a) (Lp[a]) With High-Risk Plaque and Pericoronary Inflammation Adjusted odds ratios from logistic regression models for the presence low-density plaque and presence of increased pericoronary adipose tissue attenuation (PCATa). LAD indicates left anterior descending coronary artery; OR, odds ratio; RCA, right coronary artery.

**Figure 3.**
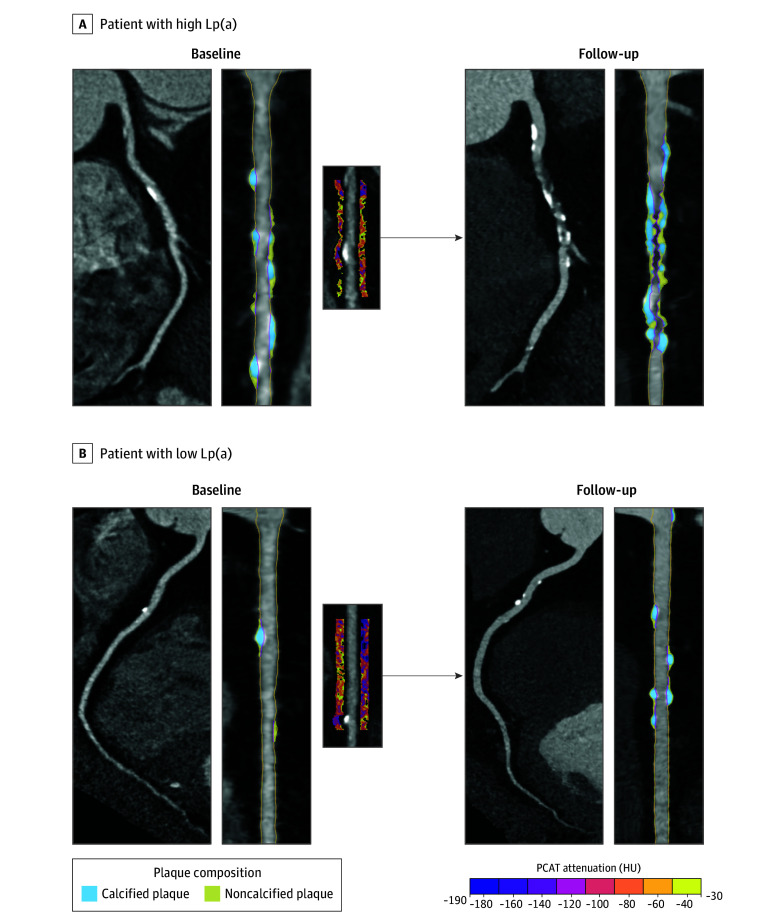
Coronary Plaque Progression and Pericoronary Inflammation With High and Low Lipoprotein(a) (Lp[a]) Two case examples of a patient with high (A) and low (B) Lp(a). Shown are baseline computed tomography angiography curved reformat (left) and straightened multiplanar reformatted (right) reconstructions of the right coronary artery (RCA) and pericoronary adipose tissue attenuation (PCATa) around the RCA. At baseline, patient A (high Lp[a]) had a percent atheroma volume (PAV) of 19.4%, a percent noncalcified plaque volume of 6.1%, and a percent noncalcified plaque volume of 13.3%, while RCA PCATa was −79.1 HU, above the scanner-specific threshold, indicative of pericoronary inflammation. During the 10-year follow-up, the plaque volumes for PAV, percent noncalcified plaque volume, and percent calcified plaque volume progressed to 30.0%, 12.2%, and 15.8% at follow-up (55% PAV increase, 100% percent noncalcified plaque volume increase, and 19% percent calcified plaque volume increase), indicative of important plaque progression. Patient B (low Lp[a]) had a baseline total plaque volume of 11.6%, an noncalcified plaque volume of 5.8%, and a calcified plaque volume of 5.8%, while RCA PCATa was −94.8 HU, below the scanner-specific threshold, indicating absence of pericoronary inflammation. During the 10-year follow-up, the plaque volumes for PAV, noncalcified plaque volume, and calcified plaque volume changed to 11.2%, 4.4%, and 6.7%, respectively (3% PAV decrease, 24% noncalcified plaque volume decrease, and 19% calcified plaque volume increase), indicative of plaque stabilization.

At baseline, 40 patients (15%) had presence of low-density plaque, which increased to 56 patients (21%) at follow-up. Every doubling of Lp(a) was associated with an adjusted odds ratio (OR) of 1.23 (95% CI, 1.00-1.51) for the presence of low-density plaque at baseline (*P* = .05; Figure 2). At follow-up, high Lp(a) levels were also associated with the presence of low-density plaque (OR, 1.21; 95% CI, 1.01-1.45 per doubling in Lp[a]; *P* = .04). Patients with higher Lp(a) levels had higher rates of increased PCATa in the univariate analysis (eFigure 3 in Supplement 1). After adjustment for clinical risk factors, every doubling of Lp(a) was associated with an OR of 1.22 (95% CI, 1.06-1.41; *P* = .01) and 1.24 (95% CI, 1.07-1.43; *P* = .004) for the presence of increased PCATa at baseline in the RCA and LAD, respectively. At follow-up, every doubling of Lp(a) was associated with an adjusted OR of 1.18 (95% CI, 1.02-1.36; *P* = .02) and 1.16 (95% CI, 1.01-1.34; *P* = .04) for the presence of increased PCATa in the RCA and LAD, respectively. Examples of baseline PCATa and coronary plaque progression in patients with low and high Lp(a) levels are shown in Figure 3.

## Discussion

In a serial CCTA cohort study with 10-year follow-up, we show that patients with high Lp(a) levels have increased coronary plaque burden at baseline and more rapid progression of coronary plaque compared with patients with low Lp(a) levels. Adjusted for clinical risk factors, total, noncalcified plaque, and calcified plaque volume associated with higher Lp(a) levels at baseline and follow-up. Furthermore, patients with high Lp(a) levels had an increased prevalence of low-density plaques and pericoronary inflammation. Collectively, these data confirm the profound impact of elevated Lp(a) levels on coronary atherogenesis of high-risk, inflammatory, rupture-prone plaques which may explain the increased risk for myocardial infarction observed in prior studies.^1,12^

Several studies have investigated the relationship between Lp(a) and coronary plaque volume or calcium. A previous study by Kaiser et al^5^ in 160 individuals undergoing repeated CCTA found increased progression of only low-density noncalcified plaque volume in patients with an Lp(a) level of 70 mg/dL (approximately 175 nmol/L) or higher compared with patients with lower Lp(a) levels. Although patients in the former cohort were more severely affected (71.5% had prior acute coronary syndrome) compared with patients in the present study, no differences in baseline plaque volume nor in overall plaque progression were observed between the patients with high and low Lp(a) levels. A post hoc analysis of 6 intravascular ultrasound trials in 3943 patients previously reported a significant association between baseline Lp(a) and total PAV.^13^ One of the trials, SATURN (Effect of Rosuvastatin Vs Atorvastatin), also investigated the relationship between Lp(a) and plaque progression, but found no difference between high and low Lp(a) patients.^14^ However, patients had similar baseline PAV and were treated with high-intensity statin therapy, while plaque composition was not evaluated and the duration of follow-up was only 2 years, which may have masked potential effects of Lp(a) on plaque progression.^14^ Studies using CACS have also reported mixed results. In an analysis from the MESA (Multi-Ethnic Study of Atherosclerosis) and Dallas Heart Study by Mehta et al,^15^ CACS were similar across different quintiles of Lp(a), while both CACS and Lp(a) were independently associated with cardiovascular events. Another study from MESA in 5975 patients undergoing repeat calcium scoring reported a slight increase in coronary artery calcium volume over 9.5 years of follow-up in patients with high Lp(a) levels.^16^ In the present 10-year follow-up study using AI-guided plaque quantification, we further substantiated the effect of Lp(a) beyond coronary calcification.

Adding to the prior findings, this study is the first to show that patients have higher overall progression of coronary plaque burden during a unique long-term follow-up of 10 years. Due to the long-term follow-up, the data provide important information about the impact of Lp(a) on the natural history of plaque progression, while the state-of-the-art analysis with atherosclerosis imaging quantitative computed tomography enabled reproducible and precise quantification of both plaque composition as well as plaque and pericoronary inflammation. These data suggest that exposure of the coronary endothelium to Lp(a) not only leads to a faster rate of coronary artery plaque progression, but also results in increased pericoronary inflammation and increased development of low-density plaque. Altogether, the present analysis may also provide rationale for clinical trials to use serial imaging with quantitative plaque imaging end points to evaluate the efficacy of Lp(a)-lowering therapies.

The mechanism by which Lp(a) causes high-risk plaque and increased rates of plaque progression has not been fully unraveled to date. Prior studies have found that the impact of Lp(a) on the vasculature and atherosclerotic lesions may be caused predominantly by oxidized phospholipids triggering inflammatory pathways and recruiting inflammatory leukocytes into the arterial wall.^3,4^ In contrast to LDL particles, which predominantly cause lipid accumulation inside coronary plaques, it has been suggested that Lp(a) directly drives coronary plaque inflammation.^17^ This would explain the observed association between Lp(a) and low-density noncalcified plaque—indicative of the inflammatory necrotic core within the plaque—in the current study. The increased development of these vulnerable coronary plaques is in line with previous results by Kaiser et al^5^ finding higher low-density plaque volume increase in patients with high Lp(a) levels during a 1-year follow-up.

We also found a persistent association between Lp(a) and PCATa during the 10-year follow-up in the present study. Previous studies have demonstrated the important prognostic value of pericoronary inflammation for major adverse cardiovascular events, even beyond detailed plaque characterization.^8,18^ In a complex bidirectional process, it is thought that proinflammatory processes in the coronary plaque and artery wall impede the maturation of adipocytes, leading to smaller, more aqueous adipocytes resulting in a higher PCATa.^19,20^ The persistent inflammation of pericoronary adipose tissue and plaque may eventually result in accelerated plaque progression leading to a higher plaque burden, but most importantly, may result in development of high-risk vulnerable, rupture-prone plaques as a cause of the observed high risk for myocardial infarction in patients with high Lp(a) levels.^1,12^

The impact of Lp(a) levels on plaque progression, low-density plaque, and pericoronary inflammation has important implications for clinical practice. The observed 0.32% increase in PAV progression per doubling of Lp(a) is of important magnitude for the long-term cardiovascular risk management. When comparing 2 similar patients, 1 with Lp(a) levels at the 10th percentile (7 nmol/L) and 1 with elevated Lp(a) levels at the 90th percentile (221 nmol/L), the patient with a high Lp(a) level would have a 1.60% (approximately 5-fold doubling) higher PAV progression over 10 years. If these 2 patients would both have an average PAV at baseline (3.3% in the current study), the patient with the high Lp(a) level would experience an additional PAV increase during a 10-year follow-up consisting of approximately 50% of their baseline PAV, underscoring the impact of Lp(a) levels on the plaque burden. Hence, the increased plaque burden and presence of low-density plaque underline the need for adequate risk lowering in patients with high Lp(a) levels. With both Lp(a)-lowering therapies in clinical trials,^1^ presence of low-density plaque and/or pericoronary inflammation in conjunction with high Lp(a) levels, even in otherwise low-risk individuals, might require prescription of these therapies to reduce cardiovascular risk to a normal range. However, prior to such a targeted treatment approach, it needs to be demonstrated what the impact of treatment is on reversibility of these imaging markers. Lastly, the lack of a strong association of Lp(a) with CACS in previous studies questions the utility of routine CACS in this patient population. Considering that up to 25% of myocardial infarctions occur in patients without coronary calcium,^21,22,23^ comprehensive plaque imaging using CCTA may facilitate identification of high-risk patients who would otherwise be left untreated.

### Limitations

The current study was a single-center study with a relatively limited sample size and fewer than half of patients undergoing baseline imaging were included in this serial analysis. Nevertheless, there were 2691 patient-years of follow-up due to the median interval of 10 years between baseline and follow-up scans. Due to this extended interval between the serial CCTA studies, scanners and protocols differed between baseline and follow-up, which may have influenced the results. Although overall PAV is unlikely to be affected by difference in scanners,^24^ plaque composition and PCATa might have been interpreted differently, despite adjustment for scanner type and settings in both the plaque and PCATa analysis. Lp(a) levels were measured at follow-up imaging. It has been established that Lp(a) levels generally are more than 90% genetically determined^1^ and oral lipid-lowering agents do not or minimally alter plasma levels. Nevertheless, several studies have shown measurement variability over time,^25,26,27,28^ although large population data showed no interaction between age and plasma Lp(a) levels in adulthood.^12^ Several patients underwent revascularization between baseline and follow-up imaging, which required exclusion of the revascularized vessels at both time points. As patients with high Lp(a) levels had increased prevalence of obstructive CAD, the magnitude of the association between Lp(a) with plaque progression might be an underestimation, although findings in the sensitivity analysis with imputation of plaque volume in missing vessels were similar. Higher baseline plaque burden due to lifetime exposure to risk factors (including Lp[a]) might have resulted in increased progression of coronary atherosclerosis, irrespective of Lp(a) levels during the study, and it remains to be determined whether reducing Lp(a) levels in patients with a high plaque burden would further reduce plaque progression. Lastly, the patients in the high Lp(a) group were slightly older than the patients with Lp(a) levels lower than 125 nmol/L. Furthermore, they were more likely to have high plasma cholesterol levels, and thus, were using statins more intensively. This difference in cholesterol levels may be the result of cholesterol carried by the Lp(a) particle, which is measured in the clinical LDL cholesterol.^29,30,31^ Although these baseline differences may have impacted the univariable analysis, consistent results were found in the multivariable analysis adjusted for cholesterol levels, as well as statin intensity at baseline and follow-up.

## Conclusions

In conclusion, using prospective serial CCTA imaging with a 10-year scan interval, we found that higher Lp(a) levels were associated with increased progression of coronary plaque burden. Furthermore, Lp(a) was associated with increased prevalence of low-density noncalcified plaque and pericoronary adipose tissue inflammation. Future studies investigating the effect of Lp(a)-lowering therapies on coronary plaque burden and pericoronary inflammation are eagerly awaited.

## Notes

Supplement 1. eMethods eTable 1. Comparison of study population to patients not undergoing CCTA at follow-up eTable 2. Unadjusted and adjusted linear mixed models for the association between Lp(a), follow-up time and plaque volume after multiple imputation eFigure 1. Flowchart of patient inclusion eFigure 2. Univariate associations between Lp(a) and plaque volumes eFigure 3. Univariate associations between Lp(a) and PCATa eReferences

Supplement 2. Data sharing statement
