# Supplementary material for: Lipoprotein(a) and Long-Term Plaque Progression, Low-Density Plaque, and Pericoronary Inflammation
Source: JAMA Cardiol. 2024 Jul 17;9(9):826–34. doi: 10.1001/jamacardio.2024.1874 (PMC11255968; doi:10.1001/jamacardio.2024.1874)
Supplement: Supplement 1. — eMethods eTable 1. Comparison of study population to patients not undergoing CCTA at follow-up eTable 2. Unadjusted and adjusted linear mixed models for the association between Lp(a), follow-up time and plaque volume after multiple imputation eFigure 1. Flowchart of patient inclusion eFigure 2. Univariate associations between Lp(a) and plaque volumes eFigure 3. Univariate associations between Lp(a) and PCATa eReferences [file jamacardiol-e241874-s001.pdf]

Supplemental Online Content

Nurmohamed NS, Gaillard EL, Malkasian S, et al. Lipoprotein (a) With Long-Term Plaque Progression, Low-Density Plaque, and Pericoronary Inflammation. *JAMA Cardiol.* Published online July 17, 2024. doi:10.1001/jamacardio.2024.1874

eMethods

eTable 1. Comparison of study population to patients not undergoing CCTA at follow-up

eTable 2. Unadjusted and adjusted linear mixed models for the association between Lp(a), follow-up time and plaque volume after multiple imputation

eFigure 1. Flowchart of patient inclusion

eFigure 2. Univariate associations between Lp(a) and plaque volumes

eFigure 3. Univariate associations between Lp(a) and PCATa

eReferences

This supplemental material has been provided by the authors to give readers additional information about their work.

© 2024 Nurmohamed NS et al. *JAMA Cardiol.*

eMethods

CCTA imaging

At baseline imaging, all patients underwent combined coronary artery calcium scoring (CACS) and CCTA using ≥64 slice CCTA scanners from the same manufacturer (Philips Healthcare, Best, the Netherlands), as described previously.[1,2] Patients were administered sublingual nitroglycerin and, if necessary, metoprolol to achieve a heart rate below 65 beats per minute. First, CACS was obtained using a noncontrast computed tomography (CT) scan. Subsequently, CCTA was performed using a 120 kV tube voltage and a prospective electrocardiogram-gated CCTA protocol triggered at 75% of the R-R interval and with automatic tracking of 100 ml iobitridol bolus (Xenetix 350, Guerbet Nederland B.V., Gorinchem, the Netherlands). At follow-up, patients also underwent CACS and CCTA using a third-generation dual source CT scanner (SOMATOM Force, Siemens Healthineers, Germany). Patients were administered sublingual nitroglycerin and, if necessary, metoprolol to achieve a heart rate below 65 beats per minute. CCTA was performed with automated tube voltage and tube current modulation (CAREKv, CAREDose 4D, Siemens Healthineers, Germany) using a prospective electrocardiogram-gated CCTA protocol triggered at 70% of the R-R interval. CCTA was performed using a weight and kV dependent contrast dose (Xenetix 350, Guerbet Nederland B.V., Gorinchem, the Netherlands) after a test bolus.

AI-QCT analysis

An artificial intelligence-based software approach was used to analyze the CCTA images (Atherosclerosis Imaging Quantitative Computed Tomography; AI-QCT; Cleerly Inc., Denver, CO).[3] This FDA-cleared software service utilizes a series of validated convolutional neural networks for image quality assessment, coronary segmentation and labeling, lumen wall

© 2024 Nurmohamed NS et al. *JAMA Cardiol.*

evaluation, vessel contour determination, and plaque characterization. Prior validation of AI-QCT has been reported in multicenter trials vs expert consensus, quantitative coronary angiography and fractional flow reserve as previously published[3–5] as well as intravascular ultrasound.[6] The algorithm first produces a coronary centerline, lumen and outer vessel wall contouring for every phase available and subsequently selects the two most optimal series for analysis. The choice for best quality image is then made on a per-vessel basis. After automated segmentation and labeling in all vessels, plaques are characterized and quantified based on the Hounsfield unit (HU) attenuation. Finally, a trained radiologic technologist provides quality assurance overview of the AI analysis.

Coronary segments with a diameter ≥1.5mm were included in the analysis using the modified 18-segment Society of Cardiovascular Computed Tomography (SCCT) model.[7] Coronary percentage stenosis was adjudicated on a per-vessel basis as per SCCT guidelines and categorized by the Coronary Artery Disease Reporting and Data System (CAD-RADS).[8] Each segment was evaluated for the presence or absence of coronary atherosclerosis, defined as any tissue structure >1 mm^2 within the coronary artery wall that was differentiated from the surrounding epicardial tissue, epicardial fat or the vessel lumen itself. Plaque volumes (mm^3) were calculated for each coronary lesion and then summated to compute the total plaque volume at the segment, vessel and patient level. At baseline, plaque volume was categorized using Hounsfield unit (HU) ranges, with low-density non-calcified plaque (LD-NCP) defined as plaques with any component on a pixel level basis and quantified on an increment of 0.1 mm^3 as <30 HU, non-calcified plaque volume (NCPV) defined as HU between -30 and +350, and calcified plaque volume (CPV) defined as >350 HU.[9] At follow-up imaging, plaque thresholds were adjusted for the different scanner and kV settings, to enable longitudinal comparison.

Pericoronary adipose tissue attenuation (PCATa) was calculated using an automated algorithm. PCAT was defined as tissue with HU ranging from -190 to -30 within a single

© 2024 Nurmohamed NS et al. *JAMA Cardiol.*

concentric layer with a radial distance from the outer vessel wall equal to the diameter of the vessel. The first millimeter of tissue following the vessel wall was excluded to prevent partial volume effects and artefacts due to contrast media in the lumen. PCATa analysis involved the proximal 10 mm to 50 mm of the vessel, excluding the first 10 mm to prevent noise from the aortic wall or left main.

© 2024 Nurmohamed NS et al. *JAMA Cardiol.*

eTable 1. Comparison of study population to patients not undergoing CCTA at follow-up

Characteristic	Overall (n = 465)	Repeat CCTA (n = 299)	No repeat CCTA (n = 166)
Age at baseline (years)	59.0±8.7	57.5±7.4	61.7±10.1
Male sex	252 (54%)	180 (60%)	72 (43%)
Hypertension	211 (45%)	130 (43%)	81 (49%)
Hypercholesterolemia	171 (37%)	117 (39%)	54 (33%)
Diabetes mellitus type 2	79 (17%)	49 (16%)	30 (18%)
BMI (kg/m^2)	27.0±4.1	27.1±4.2	26.8±4.0
Smoking history	153 (33%)	86 (29%)	67 (41%)
Family history of CAD	249 (54%)	165 (55%)	84 (51%)
Reason for referral			
Aspecific chest pain	159 (35%)	91 (31%)	68 (41%)
Atypical angina	162 (35%)	110 (37%)	52 (32%)
Typical angina	139 (30%)	94 (32%)	45 (27%)
Aspirin use	341 (74%)	216 (73%)	125 (76%)
Betablocker use	276 (60%)	181 (61%)	95 (58%)
Use of calcium antagonists	120 (26%)	77 (26%)	43 (26%)
Statin use	300 (65%)	189 (63%)	111 (67%)

Mean±SD; Median [IQR]; n (%)

© 2024 Nurmohamed NS et al. *JAMA Cardiol.*

eTable 2. Unadjusted and adjusted linear mixed models for the association between Lp(a), follow-up time and plaque volume after multiple imputation

	Unadjusted β (95% CI)	P-value	Adjusted β (95% CI)	P-value
**Percent atheroma volume**				
Lp(a), per doubling	0.66 (-0.08-1.40)	0.083	0.64 (-0.04-1.33)	0.067
Follow-up time, per 10 years	1.95 (-0.19-4.09)	0.075		
Lp(a) x follow-up time	0.52 (0.12-0.92)	0.011	0.43 (0.06-0.81)	0.026
**Percent non-calcified plaque volume**				
Lp(a), per doubling	0.41 (0.02-0.80)	0.041	0.41 (0.04-0.78)	0.029
Follow-up time, per 10 years	0.80 (-0.37-1.97)	0.179		
Lp(a) x follow-up time	0.24 (0.02-0.45)	0.035	0.20 (-0.02-0.42)	0.070
**Percent calcified plaque volume**				
Lp(a), per doubling	0.24 (-0.15-0.63)	0.233	0.23 (-0.13-0.59)	0.220
Follow-up time, per 10 years	1.10 (-0.14-2.34)	0.044		
Lp(a) x follow-up time	0.30 (0.07-0.53)	0.011	0.24 (0.03-0.46)	0.024

Lp(a), lipoprotein(a).

© 2024 Nurmohamed NS et al. *JAMA Cardiol.*

eFigure 1. Flowchart of patient inclusion

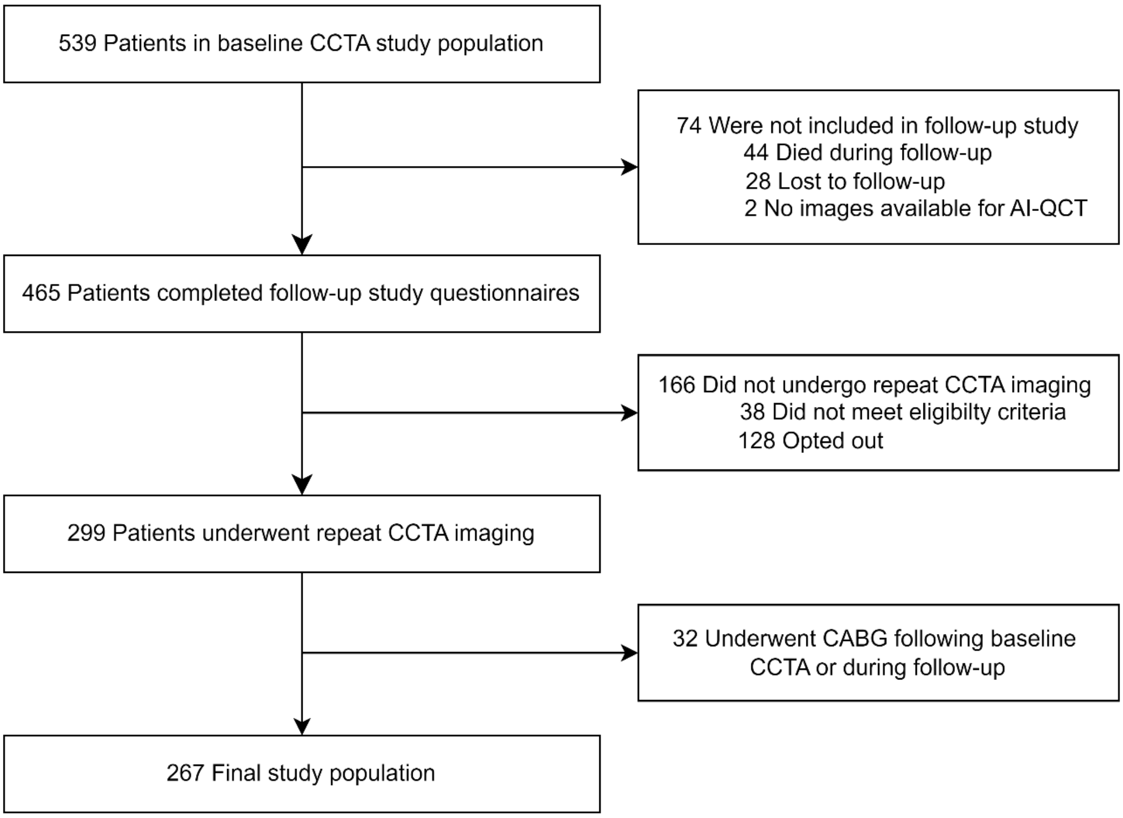

Of the baseline study population of 539 patients, 44 died during follow-up, 28 were confirmed alive through the national registry but lost to follow-up, and 2 patients had no baseline CCTA available for AI-QCT analysis. A total of 465 completed the follow-up study questionnaires after 10 years and were considered for follow-up imaging. Of these patients, 38 did not meet the eligibility criteria for repeat imaging due to various reasons: 3 had an estimated glomerular filtration rate <30 mL/min/1.73 m^2, 5 had atrial fibrillation or flutter, 5 patients were below 50 years, 3 patients were unable to provide informed consent, 2 patients had emigrated prior to the follow-up study and 20 patients had other disease prohibiting participation. A total of 128 patients were invited for follow-up imaging but opted out for the repeat CCTA. A total of 299 patients underwent repeat CCTA imaging, of whom 32 had undergone CABG between baseline and follow-up imaging (25 early revascularizations following baseline CCTA imaging, 7 late revascularizations during follow-up) and, therefore, were excluded from the current follow-up study.

© 2024 Nurmohamed NS et al. *JAMA Cardiol.*

eFigure 2. Univariate associations between Lp(a) and plaque volumes

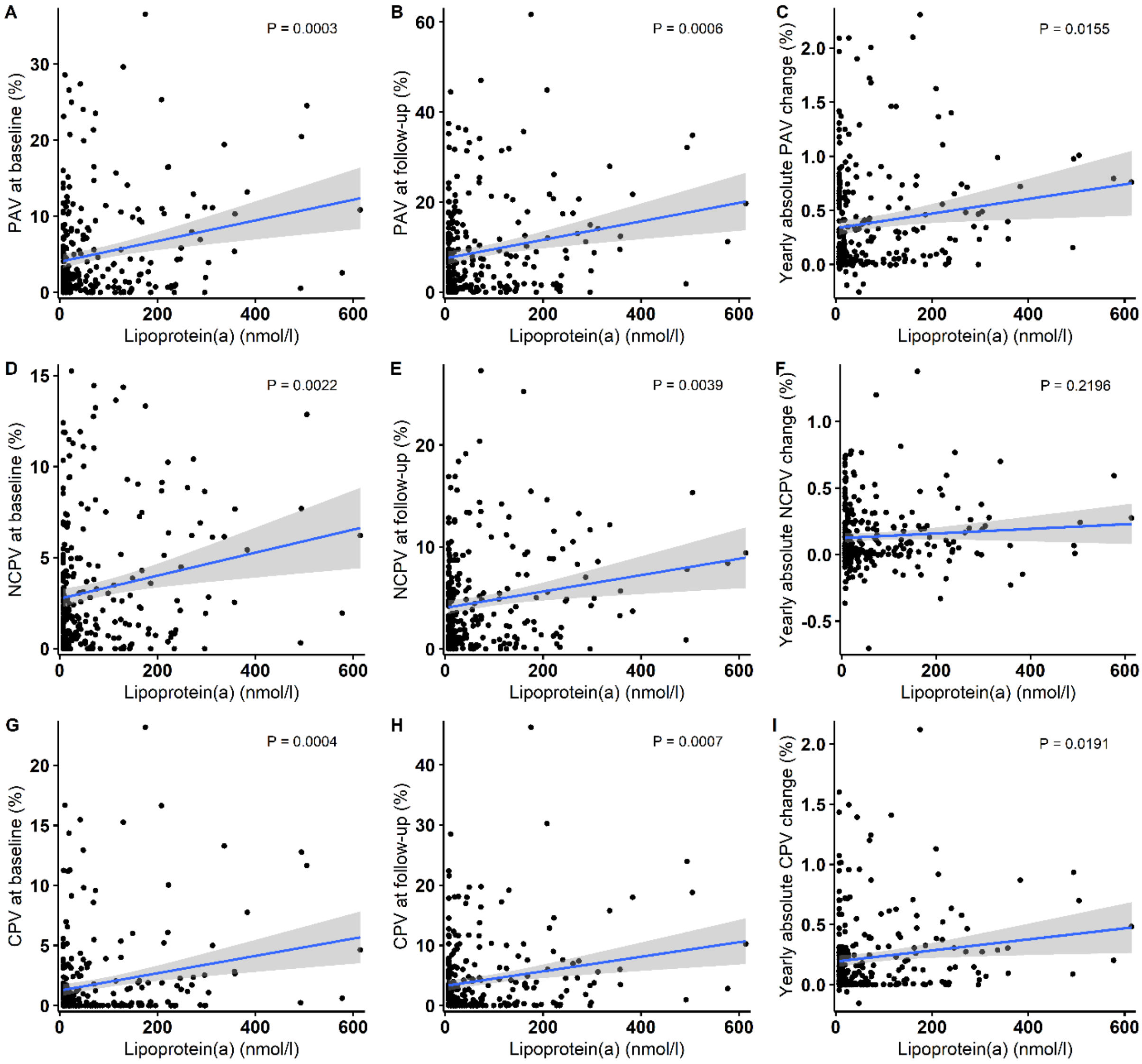

Shown is the univariate association between lipoprotein(a) and percent atheroma volume (PAV; A-C), percent non-calcified plaque volume (NCPV; D-F) and percent calcified plaque volume (CPV; G-I). Left plots illustrate plaque volumes at baseline, middle plots show plaque volumes at follow-up and right plots show the yearly change in plaque volumes during follow-up. Points represent individual patient data with a regression line with standard error and P values from univariate linear regression.

© 2024 Nurmohamed NS et al. *JAMA Cardiol.*

eFigure 3. Univariate associations between Lp(a) and PCATa

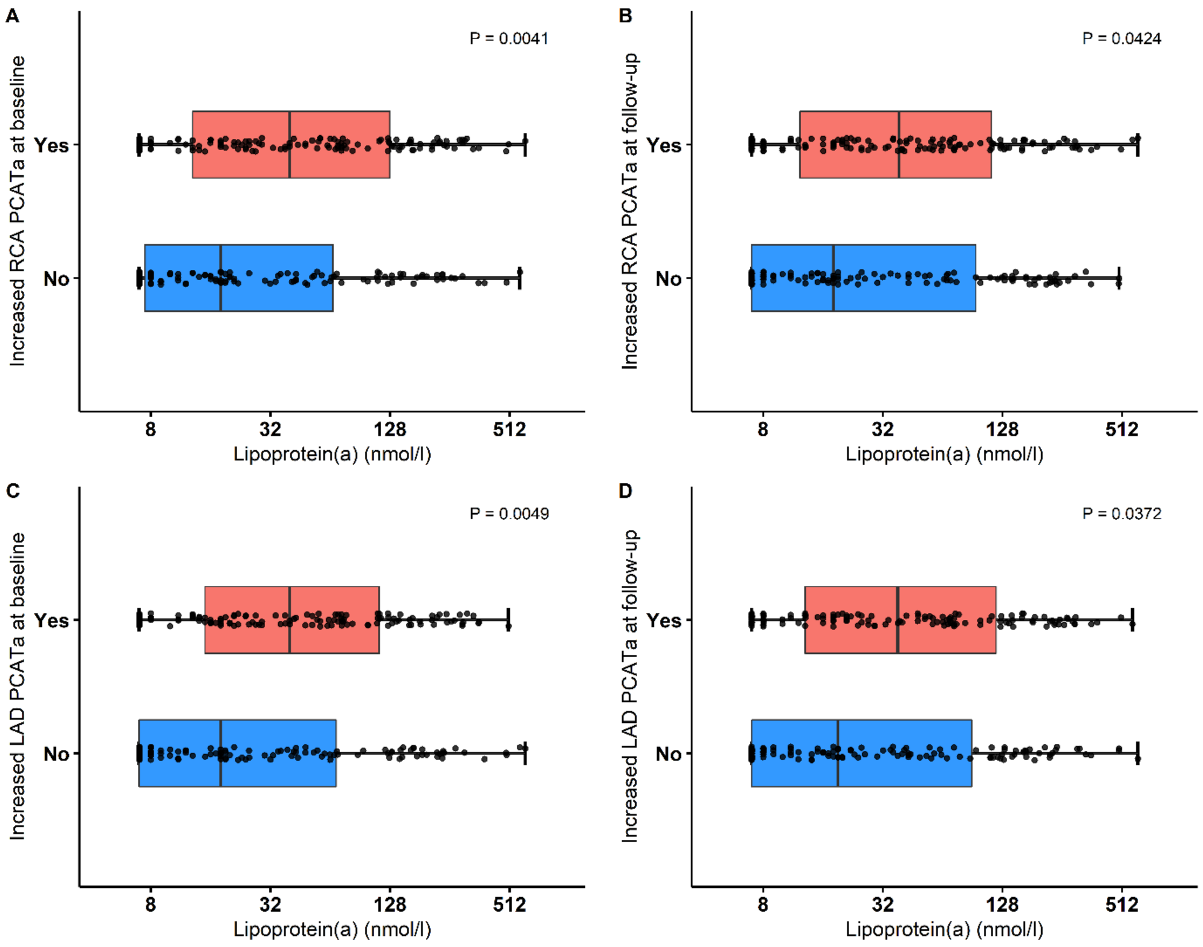

Shown is the univariate association between lipoprotein(a) and increased pericoronary adipose tissue attenuation (PCATa) in the right coronary artery (A and B) and left anterior descending coronary artery (C and D). Left plots (A and C) show baseline values, right plots (B and D) show follow-up values. Points represent individual patient data with boxplots showing the distribution. P values are from univariate logistic regression.

© 2024 Nurmohamed NS et al. *JAMA Cardiol.*

References

1. van Diemen PA, Bom MJ, Driessen RS, et al. Prognostic Value of RCA Pericoronary Adipose Tissue CT-Attenuation Beyond High-Risk Plaques, Plaque Volume, and Ischemia. *JACC Cardiovasc Imaging*. 2021;14(8):1598-1610. doi:10.1016/j.jcmg.2021.02.026

2. Nurmohamed NS, Bom MJ, Jukema RA, et al. AI-Guided Quantitative Plaque Staging Predicts Long-Term Cardiovascular Outcomes in Patients at Risk for Atherosclerotic CVD. *JACC Cardiovasc Imaging*. 2023. doi:https://doi.org/10.1016/j.jcmg.2023.05.020

3. Griffin WF, Choi AD, Riess JS, et al. AI Evaluation of Stenosis on Coronary CT Angiography, Comparison With Quantitative Coronary Angiography and Fractional Flow Reserve: A CREDENCE Trial Substudy. *JACC Cardiovasc Imaging*. 2022. doi:https://doi.org/10.1016/j.jcmg.2021.10.020

4. Jonas R, Earls J, Marques H, et al. Relationship of age, atherosclerosis and angiographic stenosis using artificial intelligence. *Open Hear*. 2021;8(2):e001832. doi:10.1136/openhrt-2021-001832

5. Choi AD, Marques H, Kumar V, et al. CT Evaluation by Artificial Intelligence for Atherosclerosis, Stenosis and Vascular Morphology (CLARIFY): A Multi-center, international study. *J Cardiovasc Comput Tomogr*. 2021;15(6):470-476. doi:10.1016/j.jcct.2021.05.004

6. Omori H, Matsuo H, Earls J, et al. Abstract 13665: Determination of Lipid-Rich Plaque by Artificial Intelligence-Enabled Quantitative Computed Tomography Using Near-Infrared Spectroscopy. *Circulation*. 2022;146(Suppl_1):A13665-A13665. doi:10.1161/circ.146.suppl_1.13665

© 2024 Nurmohamed NS et al. *JAMA Cardiol.*

7. Leipsic J, Abbara S, Achenbach S, et al. SCCT guidelines for the interpretation and reporting of coronary CT angiography: A report of the Society of Cardiovascular Computed Tomography Guidelines Committee. *J Cardiovasc Comput Tomogr*. 2014;8(5):342-358. doi:10.1016/j.jcct.2014.07.003

8. Cury RC, Blankstein R, Leipsic J, et al. CAD-RADS^{TM} 2.0 - 2022 Coronary Artery Disease – Reporting and Data System an expert consensus document of the Society of Cardiovascular Computed Tomography (SCCT), the American College of Cardiology (ACC), the American College of Radiology (ACR) and the No. *J Cardiovasc Comput Tomogr*. 2022;16(6):536-557. doi:10.1016/j.jcct.2022.07.002

9. Shaw LJ, Blankstein R, Bax JJ, et al. Society of Cardiovascular Computed Tomography / North American Society of Cardiovascular Imaging – Expert Consensus Document on Coronary CT Imaging of Atherosclerotic Plaque. *J Cardiovasc Comput Tomogr*. 2021;15(2):93-109. doi:10.1016/j.jcct.2020.11.002

© 2024 Nurmohamed NS et al. *JAMA Cardiol.*
