# Supplementary material for: Lipoprotein(a) and Long-Term Plaque Progression, Low-Density Plaque, and Pericoronary Inflammation
Source: JAMA Cardiol. 2024 Jul 17;9(9):826–34. doi: 10.1001/jamacardio.2024.1874 (PMC11255968; doi:10.1001/jamacardio.2024.1874)
Supplement: Supplement 2. — Data sharing statement [file jamacardiol-e241874-s002.pdf]

Data Sharing Statement

Nurmohamed. Lipoprotein(a) and Long-Term Plaque Progression, Low-Density Plaque, and Pericoronary Inflammation. *JAMA Cardiol*. Published July 17, 2024.
doi:10.1001/jamacardio.2024.1874

Data
Data available: No
